# Pleiotropic roles of cold shock proteins with special emphasis on unexplored cold shock protein member of *Plasmodium falciparum*

**DOI:** 10.1186/s12936-020-03448-6

**Published:** 2020-10-27

**Authors:** Ankita Behl, Vikash Kumar, Maxim Shevtsov, Shailja Singh

**Affiliations:** 1 Special Centre for Molecular Medicine, Jawaharlal Nehru University, New Delhi, India; 2 Center for Translational Cancer Research Technische Universität München (TranslaTUM), Radiation Immuno-Oncology group, Klinikum rechts der Isar, Einstein Str. 25, Munich, 81675 Germany; 3 Institute of Cytology of the Russian Academy of Sciences (RAS), Tikhoretsky ave., 4, St. Petersburg, 194064 Russia; 4 Pavlov First Saint Petersburg State Medical University, L. Tolstogo str. 6/8, St. Petersburg, 197022 Russia; 5 Almazov National Medical Research Centre, Polenov Russian Scientific Research Institute of Neurosurgery, Mayakovskogo str. 12, St. Petersburg, 191104 Russia; 6 National Center for Neurosurgery, Turan Ave., 34/1, Nur-Sultan, 010000 Kazakhstan; 7 Far Eastern Federal University, Russky Island, Vladivostok, 690000 Russia

**Keywords:** Cold shock proteins, Cold shock domain, Nucleic acid binding, YBOX-1, *Plasmodium falciparum*, Gametocytes

## Abstract

The cold shock domain (CSD) forms the hallmark of the cold shock protein family that provides the characteristic feature of binding with nucleic acids. While much of the information is available on bacterial, plants and human cold shock proteins, their existence and functions in the malaria parasite remains undefined. In the present review, the available information on functions of well-characterized cold shock protein members in different organisms has been collected and an attempt was made to identify the presence and role of cold shock proteins in malaria parasite. A single *Plasmodium falciparum* cold shock protein (*PfCoSP*) was found in *P. falciparum* which is reported to be essential for parasite survival. Essentiality of *PfCoSP* underscores its importance in malaria parasite life cycle. In silico tools were used to predict the features of *PfCoSP* and to identify its homologues in bacteria, plants, humans, and other *Plasmodium* species. Modelled structures of *PfCoSP* and its homologues in *Plasmodium* species were compared with human cold shock protein ‘YBOX-1’ (Y-box binding protein 1) that provide important insights into their functioning. *PfCoSP* model was subjected to docking with B-form DNA and RNA to reveal a number of residues crucial for their interaction. Transcriptome analysis and motifs identified in *PfCoSP* implicate its role in controlling gene expression at gametocyte, ookinete and asexual blood stages of malaria parasite. Overall, this review emphasizes the functional diversity of the cold shock protein family by discussing their known roles in gene expression regulation, cold acclimation, developmental processes like flowering transition, and flower and seed development, and probable function in gametocytogenesis in case of malaria parasite. This enables readers to view the cold shock protein family comprehensively.

## Background

Every organism faces changing environmental conditions and has evolved cellular machinery for coping with stress and adapting to changing environments. Change in temperature is one of the most common stresses faced by all living organisms. To respond to harmful effects of temperature downshift, there exists a family of proteins called cold shock proteins that play a significant role in acclimation of cells to cold [1–5]. They help the cells to adapt and have pleiotropic functions inside the cell [6, 7]. Cold shock proteins are among the most evolutionarily conserved proteins and are characterized by the presence of one or more cold shock domains (CSDs). CSDs have nucleic acid binding properties that bestow these proteins with several functions, including regulation of transcription, translation and splicing [5, 8].

At low temperatures, cold shock proteins function as RNA chaperones by destabilizing secondary structures in target RNA. This enables the maintenance of single stranded state of target RNA to pursue efficient transcription and translation [4, 9]. Cold shock proteins prevent formation of hairpin structures in RNA and, therefore, act as transcription anti-terminators [10, 11].

## Identification of cold shock proteins in bacteria

Cold shock proteins were initially found when a sudden drop in temperature (from 37 °C to 10 °C) caused a many-fold increase in the expression of a cold shock protein A (CspA) in *Escherichia coli* [12, 13]. Thereafter, cold shock proteins have been identified in several bacteria, including psychrophilic, mesophilic, thermophilic, and even hyperthermophilic bacteria [4, 13, 14]. Sequence analysis indicates that bacterial cold shock proteins are small proteins with a molecular mass of approximately 7.4 kDa [15] and comprise a typical CSD. They all have the ability to bind single-stranded RNA and DNA but no double-stranded DNA [9, 16–19]. This protein-nucleic acid interaction is mediated by the moderately well-conserved nucleic acid binding motifs RNP1 (K/R-G-F/Y-G/A-F-V/I-X-F/Y) and RNP2 (L/I-F/Y-V/I-G/K-N/G-L) [2, 20–22].

In *Escherichia coli*, several cold induced proteins are expressed that include these cold shock proteins apart from RNA helicase csdA [23], exoribonucleases PNPase and RNase R [24], initiation factors 2a and 2b, NusA and RecA [25]. Later, it was found that *Escherichia coli* encodes 9 cold shock protein genes (*CspA* to C*spI*) that share 46–91% amino acid sequence similarity [1]. Naming of cold shock proteins is done in similar fashion to other bacteria, however identical names do not necessarily share identical function and structure in different bacteria. Among all cold shock proteins, *CspC* is constitutively expressed [26] whereas *CspA*, *CspB*, *CspE, CspG*, and *CspI* are induced by cold shock [27–31]. In contrast *CspD* is induced by stationary phase growth and nutrient starvation [32, 33] and *cspF* and *cspH* expression are not linked with any particular growth condition and their functions are not known [34]. *CspC* and *CspE* are known to regulate the expression of stress response proteins ‘RpoS’ and ‘UspA’ [35] while *CspD* is implicated in persister cell formation, biofilm development and inhibits DNA replication [36, 37].

*CspA* is the major cold shock protein and the most prominent one in *Escherichia coli* [38]. A report by Giuliodori et al. suggested that mRNA of *CspA* adopts different structures at low temperature which makes it less prone to degradation [39]. As a result *CspA* mRNA is translated more efficiently upon temperature fluctuation than *CspA* mRNA at 37 °C [39]. Likewise, *ttcsp2* of thermophilic *Thermus thermophilus* was also reported to be cold induced protein that adopt more stable secondary structure in response to temperature drop [40]. At 37 °C, the *cspA* mRNA is very unstable, and has a half-life of only 12 s. Upon cold stress, its stability is dramatically increased as its half-life is now more than 20 min [41]. This transient stabilization of *cspA* mRNA on temperature drop implicates its significance in induction during cold shock [42].

Xia et al. suggested that the functions of the CspA family members overlap and can compensate for each other [31]. The authors found that by deleting 4 cold shock protein genes (*cspA, cspB, cspE, cspG*) in *E. coli*, a cold-sensitive strain ‘BX04’ was obtained that was unable to form colonies at 15 °C [31]. The cold sensitivity of this strain can be suppressed by overexpressing any of the *E. coli* cold shock protein genes, except *cspD*. Moreover, loss of one or two cold shock protein genes in *E. coli* increased the production of the remaining cold-induced cold shock protein genes. Similarly, in *Bacillus subtilis*, deletion of one or two cold shock protein genes boosted the expression of remaining cold shock proteins post-cold shock [43].

The three-dimensional structures of several bacterial cold shock proteins have been determined [44–47]. Some of these include CspA from the mesophilic bacterium *Escherichia coli* (EcCspA), cold shock protein from the thermophilic bacterium *Bacillus caldolyticus* (BcCsp) and cold shock protein from the hyperthermophilic bacterium *Thermotoga maritima* (TmCsp) [44–47]. Structural studies indicate that cold shock proteins belong to oligonucleotide/oligosaccharide-binding (OB)-fold family of proteins. OB-fold consists of 5 antiparallel beta strands that form a Greek-key beta-barrel. Knowledge of OB-folded nucleoprotein complexes was found to originate from the X-ray structures of telomere DNA-binding proteins [48–50]. Although structurally cold shock proteins are conserved, their thermo-stability differs [14, 51]. Cold shock protein of thermophilic *Thermus aquaticus* has a melting temperature of 76 °C and a rigid structure. On the contrary, CspA of the psychrotrophic *Listeria monocytogenes* has a melting temperature of 40 °C [51]. This implicates that psychrophilic cold shock proteins require higher structural flexibility to bind nucleic acids upon cold shock [14].

### Cold shock proteins in humans

The human genome encodes for about 8 members of cold shock genes namely YBX1, YBX2, YBX3, CARHSP1, CSDC2, CSDE1, LIN28A, and LIN28B [52]. The best characterized members are denoted Y-box binding protein family. The prototypic member is Y-box binding protein-1 (YB-1), encoded by the gene YBX1. Two other members of Y-box binding protein family exist namely DNA binding protein A (DbpA) and C (DbpC) that are encoded by the genes YBX3 and YBX2, respectively [52]. YB-1 was first named in 1988 to refer to transcription factors that interact with the Ybox motif in the promoter of the major histocompatibility complex class II genes [53]. YB-1 has properties of a nucleic acid chaperone and binds with both DNA and RNA. By its nuclei acid binding ability, it is involved in several mRNA- and DNA-dependent processes, including mRNA splicing, mRNA translation, DNA replication and repair [54–59]. The protein functions as a positive transcription factor to upregulate several genes, including MDR1 (multi-drug resistance-1) [58]. The MDR1 promoter activity is known to increase in response to various environmental stimuli, including anticancer agents and ultraviolet irradiation [60, 61]. YB-1 also increases resistance of cells to ionizing radiation and xenobiotics when involved in DNA repair in the nucleus [61, 62]. YB-1 nuclear localization is therefore considered an early marker of multidrug resistance of malignant cells [62–64].

Another important cold shock protein expressed in humans is calcium-regulated heat-stable protein 1 (CARHSP1); a 24 kDa protein also known as CRHSP-24. CARHSP1 binds to tumor necrosis factor (TNF) mRNA and play a role in its stabilization within P-bodies and exosomes [65]. It is dephosphorylated by calcium/calmodulin regulated protein phosphatase calcineurin [66]. CARHSP1 is a paralogue of another cold shock protein PIPPin whose expression is limited to brain cells [67, 68]. PIPPin is known to bind specifically to the 3′-UTR ends of both histone H1 and H3.3 mRNAs, encompassing the polyadenylation signal [68]. Its role is implicated in the negative regulation of histone variant synthesis in the developing brain [68]. PIPPin also interacts with other RNA binding proteins such as hnRNP A1, hnRNP K, and YB-1 [69].

A further member of human cold shock protein is known as Unr (upstream of N-ras). It was first described as upstream of N-ras and initially identified as a regulator of N-ras expression [70–73]*.* Later it was found that Unr encodes a protein that possesses 5 CSDs, and is mainly expressed in the cytoplasm [74, 75]. The gene was then renamed as CSD containing E1 (CSDE1). CSDE1 plays a key role in translational reprogramming by determining the fate of mRNAs by changing their stability and abundance [76]. CSDE1 promotes and represses the translation of RNAs and also increases and decreases their abundance. Hence the role of CSDE1 is considered bidirectional [76].

The final members of cold shock protein family in humans are denoted LIN28A or LIN28B, two highly related RNA binding proteins and proto-oncogenes [77]. The role of LIN28 is to regulate translation of mRNAs that control developmental timing, pluripotency and metabolism [78]. Besides, LIN28 is responsible for the repression of the let-7 microRNA biogenesis which is required for normal development and maintainence of the pluripotent state of cells [79–81].

### Cold shock proteins in plants

Cold shock proteins play pleiotropic functions in plants ranging from acquiring freezing tolerance to regulating embryo development, flowering time and fruit development [82]. WCSP1 is the wheat cold shock protein that was the first functionally characterized plant cold shock protein [83]. It possesses biochemical functions similar to bacterial cold shock proteins and is involved in cold adaptation. WCSP1 shows binding with both DNA and RNA and unwinds double-stranded nucleic acids in vitro and in vivo [83–85]. In response to cold stress, there is upregulation of WCSP1 mRNA and increased expression of the corresponding protein in crown tissue during prolonged cold acclimation [83]. Radkova et al*.* serologically characterized the temporal and spatial distribution of the wheat CSD proteins with regard to plant development and cold adaptation [86]. They identified 4 wheat cold shock protein genes through database analysis and classified into three classes based on their molecular masses and protein domain structures. Class I (20 kDa) and class II (23 kDa) wheat cold shock proteins were observed to accumulate in root and shoot meristematic tissues during vegetative growth. Protein expression of class I and class II wheat cold shock proteins remained high during flower and seed development. On the contrary, class III wheat cold shock protein (27 kDa) was detected only during seed development. In response to cold stress, wheat cold shock proteins accumulate in crown tissue which suggests their role in cold acclimation [86].

*Arabidopsis thaliana* has 4 cold shock proteins 1–4 (AtCSP1-CSP4), that possess an N-terminal CSD. They all show binding with RNA, single and double-stranded DNA, and are able to unwind nucleic acid duplex. AtCSP3 (At2 g17870) is the only cold shock protein that is reported to be essential for the acquisition of freezing tolerance in *Arabidopsis* [88]. Overexpression of AtCSP3 confers freezing tolerance by regulating expression of stress-related genes whose roles in freezing tolerance are not known [87]. Overexpression of AtCSP1 (CSDP1; At4g36020) is reported to delay seed germination under dehydration or salt stress conditions, whereas *AtCSP2* overexpression accelerates seed germination under salt stress [88]. Juntawong et al. reported that AtCSP1 associates with polyribo-somes via an RNA-mediated interaction. AtCSP1 is implicated in selectively chaperoning mRNAs and improved translation of ribosomal protein mRNAs during cold stress [89].

Plant cold shock proteins also regulate developmental processes. AtCSP2 is expressed many folds in meristematic tissues and ovules [90–92], and regulates flowering transition, and flower and seed development [90]. AtCSP4 (AtGRP2b; At2g21060) also plays an important role in development as *AtCSP4* overexpression leads to reduced silique length and induces embryo lethality [93].

Chaikam and Karlson characterized the cold shock proteins in rice under different stress treatments and during various stages of development [94]. The authors reported that two CSD proteins (OsCSP1 (Os02g0121100) and OsCSP2 (Os08g0129200)) in rice have nucleic acid binding activity and can complement a cold sensitive *E. coli* strain [94]. Expression of OsCSPs was found at a constant level during cold treatment that last over a period of several days. On the contrary, both OsCSP proteins and transcripts highly accumulated in reproductive tissues and tissues which exhibit meristematic activity [94]. Thus, the role of OsCSPs may be more linked with developmental processes rather than with cold tolerance.

OsCSPs are maintained at a constant level subsequent to a cold treatment lasting over a period of several days.

A time-coursed study through various stages of rice development confirmed that both OsCSP proteins and transcripts are highly accumulated in reproductive tissues and tissues which exhibit meristematic activity. CSP1 associates with polyribosomes (polysomes) via an RNA-mediated interaction.

## *Plasmodium* cold shock proteins

### Detection of cold shock protein gene in *Plasmodium falciparum* genome

**Fig. 1:**
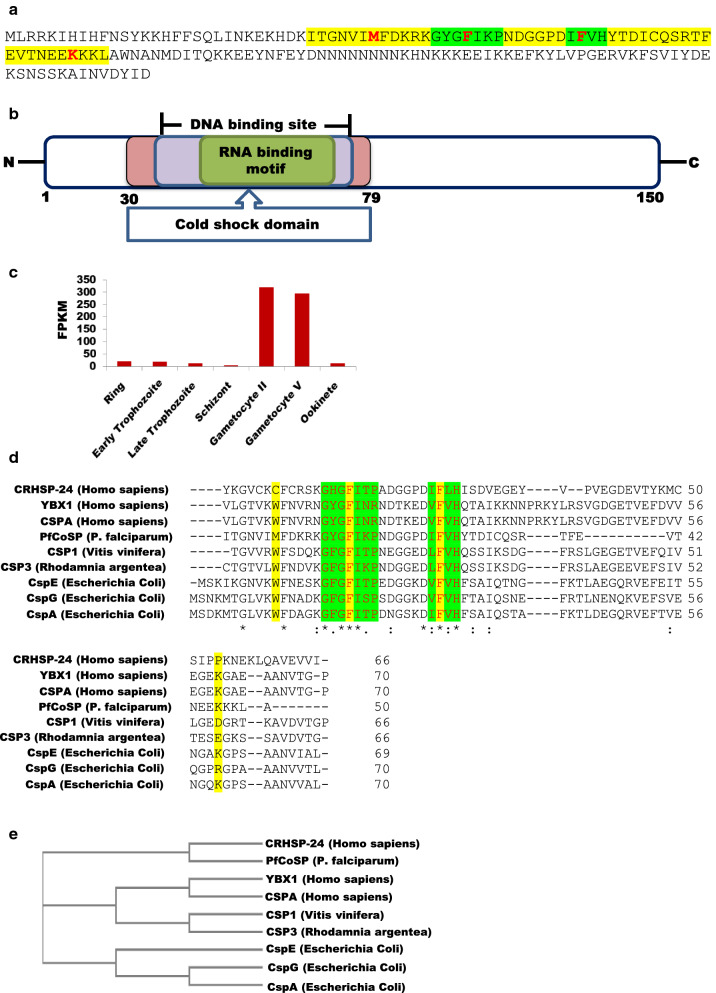
**a** Protein sequence with predicted features. Residues highlighted in yellow constitute CSD while residues in red and green denote those involved in DNA binding and RNA binding, respectively. **b** Schematic representation of domain organization and interaction sites in *PfCoSP* (**c**) Graph shows transcriptome data of *PfCoSP.* X-axis denotes *P. falciparum* 7 life-cycle stages and Y-axis represent transcript levels of fragments per kilobase of exon model per million mapped reads (FPKM). **d** Multiple sequence alignment of *PfCoSP* with its homologues in *Escherichia coli*, *Homo sapiens* and plants (*Vitis vinifera*, *Rhodamnia argentea*). Predicted residues involved in DNA and RNA binding in *PfCoSP* and their corresponding residues in other family members are marked in yellow and green bars, respectively. **e** Phylogenetic tree of *PfCoSP* with its homologues in *Escherichia coli*, *Homo sapiens* and plants (*Vitis vinifera*, *Rhodamnia argentea*)

Although there is enough information on cold shock proteins of bacteria, humans and plants but the presence of cold shock proteins and their role in the life cycle of *Plasmodium falciparum* remains largely unknown. Therefore, the existence of cold shock proteins in malaria parasites was identified and their functional relevance was explored through bioinformatics analysis. Uniprot [95] was used to search *P. falciparum* genome for presence of cold shock binding genes and a single gene was obtained (Q8I248_PLAF7), indicating the presence of cold shock protein in *Plasmodium* species. Plasmodb database [96] was then searched using its Plasmodb ID (PF3D7_0109600), and a putative protein was identified. Sequence analysis and domain organization using Blastp [97] suggested that *P. falciparum* cold shock protein (*PfCoSP*) is 150 amino acids long and harbours a typical N-terminal CSD containing DNA binding site and RNA binding motif (Fig. 1a, b). The PhenoPlasm database [98] suggested that *PfCoSP* is essential for survival of the parasite, which underlines its importance in parasite biology and makes this protein an attractive candidate for anti-malarial drug development. Transcriptome analyses from Plasmodb suggest that *PfCoSP* is upregulated at the mRNA level during gametocyte stages, suggesting the role of this protein during gametocytogenesis (Fig. 1c) [99]. However, presence of *PfCoSP* transcripts at asexual blood stages and ookinetes also hints its functional role during these stages and therefore should not be ruled out.

The presence of *PfCoSP* homologues in other species was next investigated. Organism-specific Blastp search using *PfCoSP* sequence provided its most reliable homologues in *Escherichia coli*, *Homo sapiens* and higher plants (*Rhodamnia argentea, Vitis vinifera*). Multiple sequence alignment of all sequences using Clustal omega [100] indicates similarity among cold shock protein domain only. Multiple sequence alignment was generated using the cold shock protein domain regions of all the sequences and a phylogenetic tree was constructed to investigate the sequence relationships among these cold shock proteins [100]. Predicted residues comprising the DNA binding site and RNA binding motif in *PfCoSP* were found to be moderately conserved among its homologues in bacteria, humans and plants (Fig. 1d). Phylogenetic tree suggests that *PfCoSP* and its homologues are grouped into 3 branches where *PfCoSP* was more closely related to human cold shock protein ‘CRHSP-24′ and distantly related to *Escherichia coli* cold shock proteins (CspA, CspG, CspE) (Fig. 1e).

### *Pf*CoSP homologues in other *Plasmodium* species

The presence of *PfCoSP* homologues in other *Plasmodium* species were detected using organism-specific Blastp search [97]. The amino acid sequence of *PfCoSP* was used to scan the genome sequences from two other primate parasites (*Plasmodium vivax* and *Plasmodium knowlesi*) and from 3 rodent parasites (*Plasmodium chabaudi, Plasmodium berghei* and *Plasmodium yoelii*). *PfCoSP* homologues in all these *Plasmodium* species were identified. This indicates the existence of cold shock proteins in other *Plasmodium* species apart from malaria parasites. Table 1 shows the amino acid sequence identity between *PfCoSP* and its corresponding sequence in other *Plasmodium* species.

**Table 1:** Percentage of amino acid sequence identity between *PfCoSP* and its homologues in other *Plasmodium* species

	Percentage of amino acid sequence identity				
	*P. vivax*	*P. berghei*	*P. knowlesi*	*P. chaubadi*	*P. yeolii*
*PfCoSP*	53.85	62	59.21	60.67	60.67

**Fig. 2:**
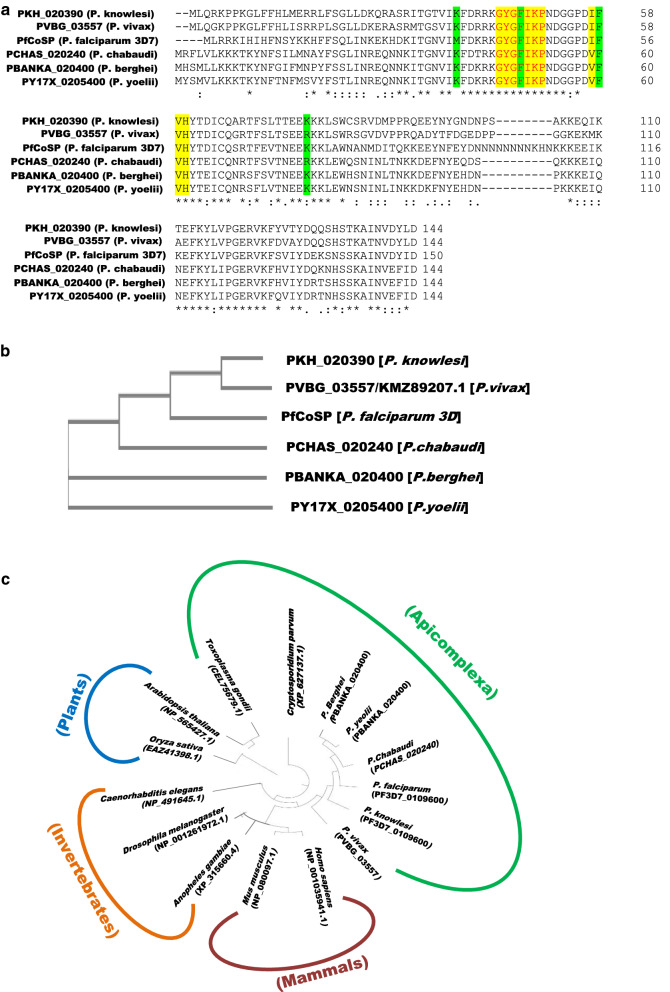
**a** Multiple sequence alignment of *PfCoSP* with its homologues in primate parasites (*Plasmodium vivax* and *Plasmodium knowlesi*) and rodent parasites (*Plasmodium chabaudi, Plasmodium berghei* and *Plasmodium yoelii*). Predicted residues involved in DNA and RNA binding in *PfCoSP* and their corresponding residues in other family members are marked in green and yellow bars, respectively. **b** Phylogenetic tree of *PfCoSP* with its homologues in other *Plasmodium* species. **c** Phylogenetic tree of the evolutionary conserved cold shock protein family. The tree was compiled using the aligned amino acid sequences of *PfCoSP* homologues from several eukaryote organisms using MEGA 6

Multiple sequence alignment showed that the predicted residues comprising the DNA and RNA binding motifs in *PfCoSP* are highly conserved among its homologues in *Plasmodium* species (Fig. 2a). A phylogenetic tree shows grouping of primate parasite homologues and rodent parasite homologues (Fig. 2b). *PfCoSP* was also found to be conserved among other protozoans, such as *Cryptosporidium parvum* and *Toxoplasma gondii*, as well as in unrelated organisms, such as *Drosophila melanogaster, Caenorhabditis elegans, Anopheles gambiae, Arabidopsis thaliana,* and *Oryza sativa* (Fig. 2c).

### In silico structural characterization of *Plasmodium* cold shock proteins

**Fig. 3:**
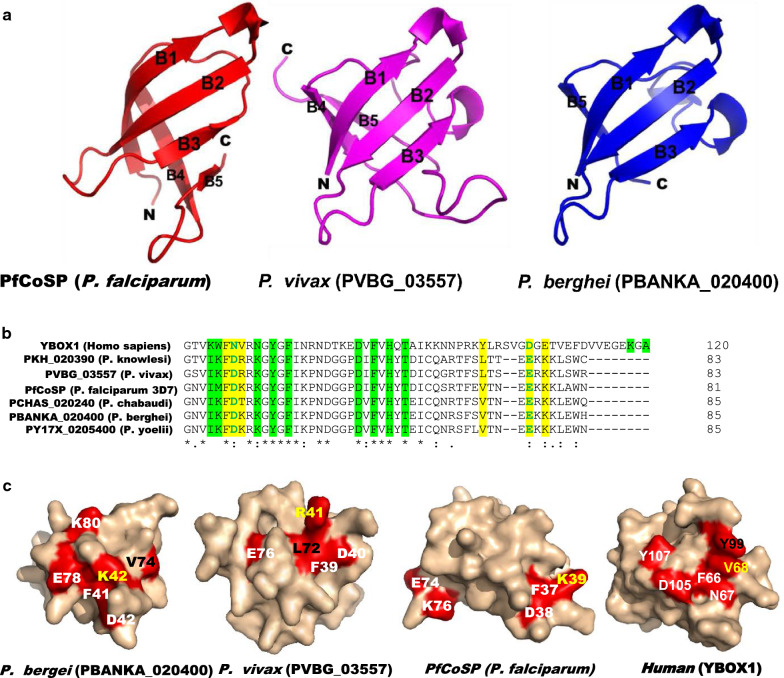
**a** Cartoon representation of homology models of cold shock protein domain of *PfCoSP* (28–73 amino acids) and its homologues in *Plasmodium vivax* (PVBG_03557, 34–102 amino acid residues) and *Plasmodium berghei* (PBANKA_020400; 36–85 amino acid residues). Five beta chains forming the oligonucleotide binding-fold are labelled. **b** Multiple sequence alignment of human YBOX-1 with *PfCoSP* and its homologues in other *Plasmodium* species. Residues known to form RNA binding site and those involved in dimerization in YBOX-1 and their corresponding residues in other cold shock protein members are marked with green and yellow bars, respectively. **c** Surface representation of human YB-1 and cold shock protein domain of *PfCoSP*, and its homologues in *Plasmodium vivax* (PVBG_03557) and *Plasmodium berghei* (PBANKA_020400). Residues responsible for human YB-1 dimerization and their corresponding residues in *PfCoSP*, *P. vivax* (PVBG_03557) and *P. berghei* (PBANKA_020400) cold shock proteins are shown in red, and are labelled. Y99 and V68 of human YB-1 and their corresponding residues that are not conserved in *PfCoSP, P. vivax* (PVBG_03557) and *P. berghei* (PBANKA_020400) are labelled in black and yellow, respectively. K78 of *P. vivax* cold shock protein (PVBG_03557) and V70 of *P. berghei* (PBANKA_020400) are obscured from view in the depicted orientation

To gain insight into the functional role of *Plasmodium* cold shock proteins, their three dimensional structures were first predicted and compared with solved structure of human YB-1. The homology modelling approach was followed to predict the structure of *PfCoSP* and its homologues in *P. vivax* and *P. berghei*. Blast search in Protein Data Bank identified the template for cold shock protein domain only for these cold shock proteins [101]. Structure of *Salmonella typhi* cold shock protein (PDB Id: 3I2Z) was used as template for modelling cold shock protein domain (28–73 amino acid residues) of *PfCoSP* while cold shock protein A from *Corynebacterium pseudotuberculosis* (PDB Id: 506F) was used for modelling cold shock protein domain of *P. vivax* (PVBG_03557; 34–102 amino acid residues) and *P. berghei* (PBANKA_020400; 36–85 amino acid residues). All the modelled structures were obtained using Modeller 9.14 [102] and refined using 3D refine [103]. 3D refine follows stepwise refinement protocol based on optimizing hydrogen bonding network and atomic-level energy minimization for improving the global and local structural quality measures [103]. Tests such as Ramachandran plot [104] and Errat [105] were run on the generated models to assess their acceptability, and were found suitable for structural analysis (Table 2). Ramachandran plot provides the stereo-chemical evaluation of backbone psi and phi dihedral angles [104] whereas Errat examines the statistics of non-bonded interactions between different atom types, and reveals the overall model quality. Generally accepted range for the good quality model is > 50. *Plasmodium* cold shock proteins were observed to comprise of OB-fold that consist of antiparallel beta strands forming beta barrel (Fig. 3a). This indicates that *Plasmodium* cold shock proteins are structurally conserved and have similar architecture as those of *E. coli* and human cold shock proteins.

**Table 2:** Ramachandran and Errat plot scores for modelled structures of *PfCoSP*, *Plasmodium vivax* (PVBG_03557) and *Plasmodium berghei* (PBANKA_020400) cold shock proteins

	*PfCoSP*	*P. vivax* (PVBG_03557)	*P. berghei* (PBANKA_020400)
Rampage^a^	96.2%	86.4%	91.5%
Errat	76.08	63.33	56.09

^a^Percentage of residues in favoured region

A recent study by Yang et al*.* solved the crystal structure of a human Y-box binding protein 1 (YB-1)-RNA complex and reveals key residues that participate in RNA binding [106]. YB-1 is a member of the CSD protein family and is recognized as an oncogenic factor in several solid tumours. It binds with RNA and plays role in several steps of post-transcriptional regulation of gene expression, including mRNA splicing, stability, and translation, microRNA processing, and stress granule assembly [106].

Multiple sequence alignment of human YB-1 with *PfCoSP* and its homologues in *Plasmodium* species was performed and found that most of the residues of human YB-1 that participate in RNA binding (K64, W65, N67, N70, Y72, F74, D83, F85, H87, T89, D105, K118, A120) are conserved in *Plasmodium* cold shock proteins (Fig. 3b). Yang et al*.* also revealed that YB-1 CSD forms a homodimer in solution, and the residues responsible for dimerization include F66, N67, V68, Y99, D105, and E107. Out of these, Tyr-99 and Asp-105 are critical for YB-1 CSD dimerization. When Tyr-99 and Asp-105 are substituted with Ala, this mutant exhibited a dramatic decrease in the interactions responsible for dimer formation. Also, it resulted in reduced RNA binding activity of YB-1, and abrogated the splicing activation of YB-1 targets. Using these data, the oligomerization state of *Plasmodium* cold shock proteins was explored. Multiple sequence alignment showed that F66 of human YB-1 was fully conserved in *Plasmodium* cold shock proteins while N67, D105, E107 were semi-conserved. However, V68 and Y99 of human YB-1 were not conserved in *Plasmodium* cold shock proteins. Since Y99 is one of the key residues for YB-1 dimerization and is not conserved in *Plasmodium* cold shock proteins, it is presumed that *PfCoSP* and its homologues in *Plasmodium* species may exist as monomeric structures. Such striking structural differences between host and parasite protein counterparts may identify essential ‘*PfCoSP*’ as a candidate for structure-based drug design against *falciparum* malaria. *PfCoSP* monomeric state, however, needs to be confirmed in vivo and in vitro to conclude this. Mapping of the residues on surface representation of human YB-1, *PfCoSP* and its homologues in *P. berghei* and *P. vivax* are shown in Fig. 3c.

### Docking of *PfCoSP* with BDNA and RNA oligo ‘UCAUGU’

**Fig. 4:**
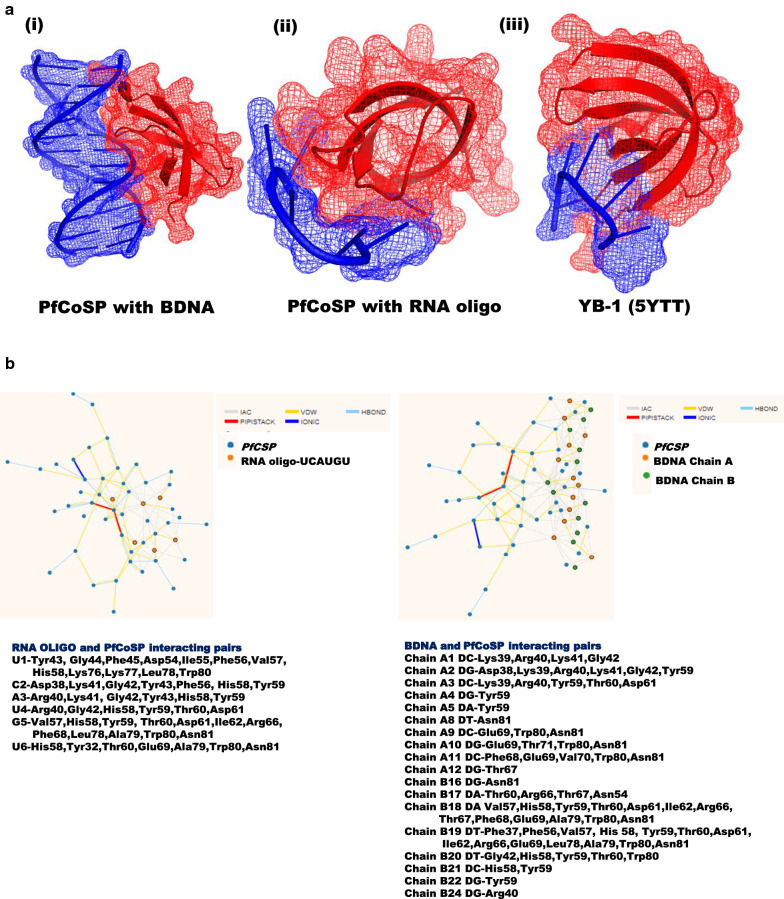
**a** Structural representation of (i) *PfCoSP*-BDNA, (ii) *PfCoSP*-RNA docked complexes and (iii) YB-1 (human cold shock protein with RNA). Red represents *PfCoSP* in i, ii (left and middle panel) and YB-1 in iii (right panel). Blue denotes BDNA in i (left panel) and RNA in ii and iii (middle and right panel). **b-i** Residue interaction network (RIN) showing the interactions between *PfCoSP* and RNA. Nodes in blue and orange on the plot represent residues of *PfCoSP* and RNA oligonucleotides, respectively. **b-ii** RIN plot of *PfCoSP*-BNA docked complex. Nodes in blue, orange and green on the plot represent residues of *PfCoSP* and BDNA A chain and BDNA B chain deoxyribonucleotides, respectively. Pairs of interactions are mentioned below the plot (**a**, **b**)

Nucleic-acid binding ability of *PfCoSP* was next investigated by docking its modelled structure with BDNA and RNA oligo ‘UCAUGU’ using PatchDock [107] and HDOCK servers [108], respectively. Docking was performed by considering the corresponding residues as active sites in *PfCoSP* that were reported RNA binding residues in YB-1 by Yang et al. [106], while no residue was given as active site in docking *PfCoSP* with BDNA. Docking energy for *PfCoSP*-BDNA and *PfCoSP*-RNA complex were observed to be −216 and −236.54 kJ/mol respectively, indicating the stability of the docked structures. *PfCoSP*-BDNA and *PfCoSP*-RNA docked complex along with that of YB-1 are shown in Fig. 4a. To identify key residues involved in interactions, the residue interaction network (RIN) profiles of docked complexes were generated using RING 2.0 web server [109]. RIN provides a visual interface to evaluate the stability of connections formed by amino acid residues at the contact sites [109]. Detail analysis of RIN plot suggested that His58 and Tyr59 of *PfCoSP* form maximum number of interactions with both BDNA and RNA. Also, Phe45 and Phe56 were greatly involved in forming the network of *PfCoSP* with RNA. These results are coherent with a previous report where corresponding residues of His58, Phe45 and Phe56 in YB-1 were reported to be critical for RNA binding by the CSD of YB-1 [106]. Various interactions in the form of a detailed network model are represented in Fig. 4b.

### Functional aspects of *PfCoSP*

An attempt was made to explore the functional aspects of *PfCoSP* in the malaria parasite. In silico analysis was performed using the MEME suite tool [110] to identify motifs that can be correlated with the functional role of *PfCoSP* in malaria parasites. The search identified 3 motifs ‘WNANMDITQ’, ‘LRRKIH’ and ‘HFNSYK’ in *PfCoSP*. These motifs were searched in protein sequence databases by using Find Individual Motif Occurrences (FIMO) [111]. Site-specific DNA methytransferases, phosphatidylinositol 4-kinases and homeobox protein were found as significant matches for motifs WNANMDITQ, LRRKIH and HFNSYK, respectively (Table 3). Interestingly, all these proteins/enzymes are DNA or nucleotide-binding proteins. DNA methytransferases and homeobox proteins play a key role in regulating gene expression while phosphatidylinositol 4-kinases modulate inter-organelle lipid trafficking and phosphoinositide signalling [112–114]. These in silico data hint that *PfCoSP* may have a role in controlling gene expression and regulating lipid trafficking by its nuclei acid binding ability.

**Table 3:** Identified motifs in *PfCoSP* using MEME tool and their matched sequences found in protein sequence database by find individual motif occurrence (FIMO)

	MOTIF LOGO	Matched sequence by FIMO	Protein family	Biological function
1		WNFAYDITQ	Site specific DNA methytransferases	They catalyze the transfer of a methyl group to DNA. DNA methylation is one of several epigenetic mechanisms that cells use to control gene expression
2		LVRGIR	Phosphatidylinositol 4-kinases	Catalyzes phosphorylation of phosphatidylinositol (PI) at the D-4 position, yielding phosphatidylinositol 4-phosphate (PI4P), a lipid that plays important roles in Golgi function, protein sorting and membrane trafficking. Besides, phosphorylation of PI to PI4P is the first step in the generation of phosphatidylinositol 4,5-bisphosphate (PIP2), a precursor of the second messenger inositol 1,4,5-trisphosphate
3		HFNRYL	Homeobox proteins	Homeobox genes encode homeodomain protein products that bind DNA to regulate expression of target genes

Significant matches of proteins/enzymes for these motifs are mentioned along with their biological function

### Parasite life cycle and *PfCoSP*

Complex and multi-stage life cycle of the malaria parasite involves two hosts, humans and female *Anopheles* mosquitoes. The majority of circulating parasites in an infected human are asexually dividing merozoites [115]. A small portion of these undergo a differentiation pathway and a series of changes, which lead to the generation of a sexually competent parasite called ‘gametocyte’ [116]. This maturation is called gametocytogenesis which involves formation of pre-gametes that later fertilize in the mosquito to complete the sexual cycle. Transmission from an infected human host to a susceptible mosquito occurs through these highly specialized gametocytes. After a mosquito feeds on an infected host, gametocytes egress from their host erythrocytes to initiate gametogenesis within the mosquito midgut lumen. Female gametocytes produce a single non-motile spherical female gamete, while male gametocytes undergo ‘exflagellation’, a process that results in the production of 8 motile male gametes [116].

*As P. falciparum *gametocytes switch from human host to female *Anopheles*, it results in transitioning from the relatively protected environment within the human erythrocytes to being an exposed parasite in the lumen of a mosquito. Apart from the fact that the parasite becomes extracellular, there is a modified pH and, importantly, drop in temperature [116]. It is obvious that the parasite prepares itself for upcoming environmental change. Transcriptome analyses suggest that ~ 300 genes are upregulated at the mRNA level during gametocyte development [117, 118]. In silico analysis identified a cold shock protein in malaria parasite (*Pf*CoSP) whose transcriptome data also suggest that it is upregulated at gametocyte stages [99]. *Pf*CoSP is reported to be essential for parasite survival [98]. This hints that *PfCoSP* may play its essential role in egress machinery of gametocytes by regulating gene expression of pivotal genes that are involved during gametocytogenesis. Apart from the gametocyte stages where the role of *Pf*CoSP can be related to temperature difference phenotype, *Pf*CoSP transcripts are also found at ookinete and asexual blood stages of malaria parasite. This further suggests that *Pf*CoSP may play its functional role at other stages of parasite life cycle.

## Conclusions

This review highlights the significance of cold shock proteins in several organisms and sheds new light on the unexplored cold shock protein member of *P. falciparum*. Functional diversity of cold shock protein family is entailed by discussing their known roles in gene expression regulation, cold adaptation, disease progression, and developmental processes such as flowering transition and flower and seed development. In silico work described here provide structural information of *PfCoSP* and hints its functional role particularly in regulating gene expression at gametocyte stages. However, the presence and structure–function characterization of *PfCoSP* needs to be confirmed in vivo and in vitro to conclude its role in gametocytogenesis and also at other stages of parasite life cycle. Future studies should aim at the structural and functional characterization of *PfCoSP* to understand its pivotal role in malaria parasites. Since cold shock proteins are verifiable targets for therapeutic intervention, the work described here, along with future studies on *PfCoSP*, may help with strategies aimed at targeting this protein directly for the development of anti-malarials and transmission-blocking vaccines.

## Data Availability

Not applicable.

## Abbreviations

CSD: Cold shock domain
YBOX-1: Y-box binding protein 1
*PfCoSP*: *Plasmodium falciparum* Cold shock protein
CARHSP1: Calcium-regulated heat-stable protein 1
MDR1: Multi-drug resistance-1
